# Immunoglobulin G: A useful outcome marker in the follow‐up of cystic fibrosis patients?

**DOI:** 10.1002/iid3.426

**Published:** 2021-03-30

**Authors:** Laurence S. Hanssens, Sarah Cellauro, Jean Duchateau, Georges J. Casimir

**Affiliations:** ^1^ Department of Pediatric Pulmonology and Cystic Fibrosis Clinic, Hôpital Universitaire des Enfants Reine Fabiola Université Libre de Bruxelles (ULB) Brussels Belgium; ^2^ Laboratoire Académique de Pédiatrie, Hôpital Universitaire des Enfants Reine Fabiola Université Libre de Bruxelles (ULB) Brussels Belgium

**Keywords:** children, cystic fibrosis, hypergammaglobulinemia, hypogammaglobulinemia, immunoglobulins

## Abstract

**Background and Methods:**

Hypergammaglobulinemia (hyper‐IgG) and hypogammaglobulinemia (hypo‐IgG) have been reported in patients with cystic fibrosis (CF). Although the clinical respiratory course is paradoxically different, depending on the IgG status, this association remains elusive. Therefore, we performed a longitudinal study to assess the annual evolution of IgG profiles in a cohort of pediatric patients with CF, from their diagnosis until 2016. We then compared clinical findings with the patients’ IgG status to determine whether IgG status could reflect the respiratory clinical course of patients with CF.

**Results:**

Among the 66 patients with CF that were aged between 12 months and 18 years in 2016 (mean age: 9.3 years [SD: 5.2]), hypo‐IgG was observed in 15.2% and no hyper‐IgG was identified. Longitudinal assessment since diagnosis revealed no hyper‐IgG but 33.3% of patients had at least one sample showing hypo‐IgG, among which two patients displayed persistent hypo‐IgG. The number of pulmonary exacerbations, duration of antibiotic therapy, and erythrocyte sedimentation rate were all lower in hypo‐IgG patients. No difference was observed for the genotype, chronic *Pseudomonas aeruginosa* or *Staphylococcus aureus* infection, and in the parameters of lung function.

**Conclusion:**

The IgG profile of pediatric patients with CF has changed over recent decades, particularly with regard to hyper‐IgG. In a significant portion of the pediatric CF population, hypo‐IgG is transient and only identifiable in longitudinal assessments. This study reinforces that hypo‐IgG patients paradoxically present a more favorable course of clinical status. Therefore, IgG levels could be a useful outcome marker in the follow‐up of patients with CF.

## Highlights

• Abnormal immunoglobulin G (IgG) levels have been described in patients with cystic fibrosis (CF). With this longitudinal study, we sought to determine whether IgG status could reflect the respiratory clinical course of CF patients. The assessment in 2016 identified 15.2% of 66 pediatric patients as hypo‐IgG, whereas no hyper‐IgG was highlighted. The longitudinal assessment of IgG since diagnosis revealed no hyper‐IgG and hypo‐IgG was transient in at least one‐third of our patients, except for two, who both displayed persistent hypo‐IgG. Therefore, three populations can be identified, according to the patient's IgG profile during their follow‐up: a first with persistent hypo‐IgG since diagnosis; a second with a transient deficiency, which likely normalizes in adolescence; a third whose IgG remains within a normal range. CF patients with hypo‐IgG appear to be protected from pulmonary exacerbations and therefore could present a more favorable respiratory course, even with low IgG levels since diagnosis. This suggests that IgG levels could be a useful outcome marker in CF patient follow‐up in order to predict their respiratory clinical course.

## 1 INTRODUCTION

Cystic fibrosis (CF) is a recessive genetic disease resulting from mutations in the CF transmembrane conductance regulator (CFTR) gene, which exhibits pathologic consequences in a number of organs. However, pulmonary disease remains the main cause of morbidity and mortality, which is characterized by recurrent cycles of obstruction, infection, and inflammation that lead to bronchiectasis and progressive pulmonary injury.^1^, ^2^

Abnormal immunoglobulin (Ig) levels, particularly IgG levels, have been described in patients with CF.^3^, ^4^, ^5^, ^6^, ^7^, ^8^, ^9^, ^10^, ^11^ Hypergammaglobulinemia (hyper‐IgG) is the consequence of chronic respiratory infection and inflammation. The typical CF pathogens are *Staphylococcus aureus* (SA) and *Pseudomonas aeruginosa* (PA) because they can adapt within the CF lung. High IgG levels are associated with worse clinical‐status courses and lung function, suggesting a connection with the severity of lung disease.^3^, ^5^, ^6^ Conversely, hypogammaglobulinemia (hypo‐IgG) is also reportedly associated with less‐severe lung disease and thus better prognosis.^3^, ^5^, ^6^ Abnormal IgA or IgM levels, or common variable immune deficiency, have also been described in CF.^5^ Abnormalities in the systemic immune response to parenteral immunization, immunoglobulin metabolism, or specific T‐ and B‐cell function have not been well identified.

The mechanisms of these paradoxical associations between IgG status and respiratory clinical course remain unknown. Despite marked progress in understanding the CF pathogenesis, the link between mutations in the CFTR gene and manifestations of the disease is not completely understood. It is clear that other factors, in addition to CFTR, modulate the pathology and course for patients with CF.^12^ One such factor could be IgG status. However, these studies cited above are not particularly longitudinal and some are relatively old. Therefore, it is likely that they no longer reflect the immunological status of patients that have benefited from optimal management in recent decades, which has significantly improved both the quality of life and life expectancy of patients with CF.

This longitudinal study assesses the annual evolution of IgG profile and clinical findings in a cohort of pediatric patients, from their diagnosis until 2016. In so doing, we sought to determine whether IgG status could reflect the respiratory clinical course of patients with CF.

## 2 METHODS

### 2.1 Study design and patients

This is a retrospective study that includes all pediatric patients with CF attending the CF center of the *Université Libre de Bruxelles* in 2016. Sixty‐six patients aged from 12 months to 18 years were considered. These patients’ IgG levels were measured using nephelometry at annual assessment, from their diagnosis until 2016. We converted these levels into Z‐scores which correspond to the deviation from the mean and are expressed in standard deviation. Hypo‐IgG and hyper‐IgG were respectively defined when the values of a normal age‐matched population were below percentile = 0.025 (corresponding to a Z‐score < −1.96) and above *p* = .975 (corresponding to a Z‐score ≥  + 1.96).

### 2.2 Clinical findings

The minimum frequency of examinations for the patients with CF was quarterly visits to the center, since their diagnosis. Clinical and lung function parameters were collected for each patient at each routine visit. The cumulative number of pulmonary exacerbations (PEx) and duration of antibiotic therapy (AB) were defined during the last 12 months preceding the assessment of IgG. PEx was defined as an acute episode requiring antibiotic therapy by any means of administration. Nutritional status was expressed as the mean annual values of body mass index Z‐score (BMI Z‐score), which was adjusted for age and gender according to the Centers for Disease Control and Prevention guidelines.^13^ Lung function tests were performed in line with the American Thoracic Society and European Respiratory Society guidelines.^14^ The mean annual values of forced vital capacity (FVC), forced expiratory volume in one second (FEV_1_), and forced expiratory flow 25%–75% (FEF_25–75_) were also collected, which were expressed as percentages of the normal predicted values for age, gender, and height.^15^ Inflammatory markers (neutrophils, C‐reactive protein [CRP], and erythrocyte sedimentation rate [ESR]) were also performed during annual assessment.

This study was approved by our hospital's ethics committee (CEH no. 06/17).

### 2.3 Statistics

All data were tabulated with Microsoft Office® Excel® 2016 (32‐ & 64‐bit) software (Zaventem, Belgium) and statistics were completed using the statistical Analyse‐it® software (Version no. 4. 81.6) (Analyse‐it Software Ltd.). IgG levels and normal laboratory values, according to the age categories (http://www.chu-brugmann.be:88/sitesq/appPublic/modANL/ANL-itemFrame.asp?oid=ANL000614%26objstatus=dft%26frompg=ANL-itemlist.asp), were transformed into logarithmic values and then smoothed by polynomial regression and transformed into Z‐scores which were adjusted for age. The algorithms employed to incorporate the age range of 4–240 months are detailed in Supporting Information. The Mann–Whitney test, Pearson's *χ* ^2^ tests, Fisher's exact test, Spearman's rank correlation and one‐way analysis of variance with repeated measures were employed.

## 3 RESULTS

### 3.1 Clinical findings and IgG profile in 2016

The clinical findings of the CF cohort in 2016 are shown in Table 1.

**Table 1:** Clinical findings of the cystic fibrosis cohort in 2016

Clinical findings	*N* = 66
Mean age, years (*SD*)	9.3 (5.2)
Gender ratio, *n* (M/F)	33/33
Genotype	
Severe, *n* (%)	47 (71.2)
F508del/F508del, *n* (%)	24 (36.4)
Median BMI Z‐score (IQR)	−0.6 (1.7)
Median FEV1% (IQR) (*n* = 51)	88.4 (17.2)
Median FEF_25%–75__%_ (IQR) (*n* = 51)	69.0 (39.5)
Chronic *PA, n* (%)	9 (13.6)
Median number of PEx (IQR)	1.5 (2.1)
Median duration of AB, days (IQR)	19.5 (37.1)
Median PMNs/mm^3^ (IQR)	3600 (2103)
Median CRP, mg/L (IQR)	0.6 (1.2)
Median ESR, mm/h (IQR)	11.0 (12.1)

*Note*: Data have been expressed as mean ± standard deviation, as median and interquartile range, or as percentages.

Abbreviations: AB, antibiotics; BMI, body mass index; CRP, C‐reactive protein; ESR, erythrocyte sedimentation rate; FEV1%, forced expiratory volume in percentage of predicted value; FEF_25%–75%_, forced expiratory flow 25%–75% as percentage predicted value; Gender ratio (M/F), male/female; PA, *Pseudomonas aeruginosa*; PEx, pulmonary exacerbations; PMNs, polymorphonuclear neutrophils.

Hypo‐IgG was observed in 10 patients (15.2%) while no hyper‐IgG was identified. Among the 36 patients that were younger than 10 years old, eight patients (22.2%) exhibited hypo‐IgG whereas only two patients (0.7%) of 30 patients aged 10–18 years had hypo‐IgG. Our results have been compared with those of previous studies (Table 2).

**Table 2:** Prevalence of hypo‐IgG and hyper‐IgG in previous and present studies

Studies	No. of patients	Age (years)	Hypo‐IgG (%)	Hyper‐IgG (%)
Wallwork, 1974	40	0.06–23	7.5	10.0
Shakib, 1976	16	0.4–17	6.3	31.3
Turner, 1978	106	0.3–18	5.7	11.3
Matthews, 1980	332	0–20	10.8	16.3
	154	< 10	22.0	6.5
	178	10–20	1.0	24.7
Shryock, 1986	19	1–31	0	10.5
Moss, 1987	31	5–41	3.2	61.0
Hodson, 1988	32	17–49		69.0
Pressler, 1988	126	13^a^ (1–36)		32.0
Abman, 1991	42	2.3^a^	0	0
Hassan, 1994	13	14–41		69.0
Garside, 2005	154	9.2 (0.3–18)	1.9	7.8
	83	< 10	2.4	1.2
	71	10–18	1.9	15.5
Proesmans, 2011	73	10^a^ (5–15)	2.7	16.4
Present study, 2016	66	9.7^a^ (1–18.5)	15.2	0
	36	< 10	22.2	0
	30	≥ 10	0.7	0

*Note*: From Garside et al.^3^

^a^ Median age.

### 3.2 Clinical findings in the hypo‐IgG group and comparison with normo‐IgG group in 2016

As the median age significantly differed between hypo‐IgG and normo‐IgG patients (5.1 years [IQR: 5.8] vs. 10.2 years [IQR: 7.8]; *p* = .03) in 2016, we performed a comparison matched for gender and age (Table 3). Furthermore, to avoid any reduced effectiveness, we selected two paired controls for one patient with hypo‐IgG. The number of PEx and duration of AB therapy, as well as ESR, were lower in hypo‐IgG patients. These patients also displayed a significant positive correlation with the IgG Z‐score (*r*_s_ = 0.366, *p* < .005; *r*_s_ = 0.419, *p* < .001; *r*_s_ = 0.433, *p* < .001, respectively). No difference was observed for the genotype and chronic *PA* or *SA* infection, nor in the BMI, lung function parameters, or other inflammatory markers (Table 3).

**Table 3:** Clinical findings in hypo‐IgG group and comparison with normo‐IgG group matched for gender and age in 2016

	Hypo‐IgG	Normo‐IgG	
Clinical findings	(*N* = 10)	(*N* = 20)	*p*
Mean age, years (SD)	6.0 (5.2)	6.2 (4.8)	.933
Gender ratio, *n* (M/F)	5/5	10/10	1
Genotype			
Severe, *n* (%)	8 (80.0)	17 (85.0)	.729
F508del/F508del, *n* (%)	5 (50.0)	10 (50.0)	1
Median BMI Z‐score (IQR)	−0.1 (1.7)	−0.4 (1.9)	.631
Median FEV1% (IQR)	104.4 (17.2)	92.1 (21.7)	.535
	(*n* = 6)	(*n* = 11)	
Median FEF_25%–75__%_ (IQR)	91.2 (33.6)	79.4 (36.9)	.779
	(*n* = 6)	(*n* = 11)	
Chronic *PA, n* (%)	1 (10.0)	3 (15.0)	.704
**Median number of PEx (IQR)**	**1.0 (1.5)**	**2.0 (2.0)**	**.022**
**Median duration of AB, days (IQR)**	**3.5 (17.0)**	**24.5 (28.0)**	**.011**
Median PMNs,/mm^3^ (IQR)	3 360 (2 610)	5 240 (4 150)	.059
Median CRP, mg/L (IQR)	0.5 (8.8)	0.5 (1)	1
**Median ESR, mm/h (IQR)**	**2.5 (2)**	**9.0 (12)**	** < .001**

*Note*: Data have been expressed as mean ± standard deviation, median, and interquartile range, or percentages; a *p* value of  ≤ .05 was considered statistically significant.

Abbreviations: AB, antibiotics; BMI, body mass index; CRP, C‐reactive protein; ESR, erythrocyte sedimentation rate; FEV1%, forced expiratory volume in percentage of predicted value; FEF_25%–75%_, forced expiratory flow 25%–75% as percentage predicted value; Gender ratio (M/F), male/female; PA, *Pseudomonas aeruginosa*; PEx, pulmonary exacerbations; PMNs, polymorphonuclear neutrophils.

### 3.3 Longitudinal profile of IgG since diagnosis

Longitudinal assessment of annual IgG Z‐scores since diagnosis revealed a progressive increase in mean IgG Z‐scores among the CF cohort, according to the age groups (*p* = .001; Table 4).

**Table 4:** Progressive increase in mean IgG Z‐scores depending on the age groups in the CF cohort

Age	0–1	2–3	4–5	6–7	8–9	≥ 10
Years	(*N* = 54)	(*N* = 74)	(*N* = 77)	(*N* = 66)	(*N* = 61)	(*N* = 110)
Mean Z‐scores (*SD*)	−1.27 (1.54)	−0.56 (1.31)	−0.52 (1.58)	−0.30 (1.19)	−0.27 (1.23)	−0.13 (1.28)

*Note*: *N* = number of samples.

Abbreviations: CF, cystic fibrosis; *SD*, standard deviation.

The mean IgG Z‐score since diagnosis of the 10 hypo‐IgG patients in 2016 was significantly lower than that of the 20 normo‐IgG control patients (−2.4 [SD: 0.6] vs. −0.5 [SD: 0.8]; *p* < .0001).

Since diagnosis, 442 samples were collected among the CF cohort with an average of eight samples per patient. No hyper‐IgG was observed. However, we identified 22 patients (33.3%) with at least one sample showing hypo‐IgG since their diagnosis and 44 patients (66.7%) without hypo‐IgG. Two patients aged 10 and 18 years old in 2016 displayed persistent hypo‐IgG and were excluded from the complementary analysis. These patients had normal IgA and M levels. Among the patients with transient hypo‐IgG (*N* = 20), the number of samples showing hypo‐IgG was a mean of 1.9 (SD: 0.9). No significant difference was found for the age in 2016 between the patients with transient hypo‐IgG and those without hypo‐IgG (7.9 years [SD: 5.5] vs. 9.7 [SD: 5.5]; *p* = .228). However, hypo‐IgG was observed more often in children aged ≤ 10 years old than those > 10 years old (11.0% vs. 0%; *p* = .0006). In the patients with transient hypo‐IgG, the mean IgG Z‐score was −1.61 (SD: 1.06) since diagnosis, whereas the mean IgG Z‐score was 0.01 (SD: 0.68) in the patients without hypo‐IgG (*p < *.0001). Compared to patients without hypo‐IgG, the duration of AB therapy (8.5 vs. 23.0 days; *p* = .015) and ESR (4 vs. 14 mm/h; *p* < .00001) collected in 2016 were lower in the patients with transient hypo‐IgG. The patients with persistent hypo‐IgG (*N* = 2) had no PEx in 2016.

## 4 DISCUSSION

In the present study, the assessment of IgG profile in 2016 identified 15.2% of our pediatric cohort as hypo‐IgG, whereas no hyper‐IgG was highlighted. A comparison with previous studies reveals heterogeneity of hypo‐IgG percentages, which may be due to the observation that most of these studies have included both pediatric and adult patients. Matthews et al.^5^ have reported a total hypo‐IgG prevalence of 10.8%, reaching 22% in the age class under 10 years, in line with the current study. However, the longitudinal assessment of IgG Z‐scores since diagnosis demonstrated that hypo‐IgG was transient in a significant portion of our cohort, concerning at least one‐third of patients, except for two, who both displayed persistent hypo‐IgG. In their 5‐year longitudinal study, Wheeler et al.^6^ reported that 21.4% of their patients had persistent hypo‐IgG and 24.3% had initial hypo‐IgG. This observation of transient hypo‐IgG can therefore also explain differences in the percentage of hypo‐IgG among studies.

Therefore, pediatric CF patients appear to be classifiable into three populations, according to their IgG profile: a first population with persistent hypo‐IgG since their diagnosis; a second population with a transient deficiency, which appears to be more common in children under 10 years and appears to no longer be observed after this period; a third population whose IgG remains in the normal range. The progressive increase in mean IgG Z‐scores since diagnosis, according to age group, supports immunological maturation increases with age, thereby possibly explaining transient deficiency in patients with CF.^16^, ^17^, ^18^

A comparison of the hypo‐IgG patients and normo‐IgG controls in 2016 reveals that the mean IgG Z‐score since diagnosis was significantly lower in the hypo‐IgG patients, as also seen in the patients with transient hypo‐IgG. Despite their IgG levels remaining below normal since diagnosis, the CF patients with hypo‐IgG did not display a higher number of PEx. On the contrary, they even seemed protected, as evidenced by the lower number of PEx and duration of antibiotics. In addition, they appeared to exhibit less‐significant inflammation, as demonstrated by their ESR values. Indeed, hypo‐IgG has been associated with less‐severe lung disease and thus better prognosis.^3^, ^5^, ^6^ The mechanisms of this paradoxical association remain unknown. Neither age nor gender influences this association, as demonstrated in this present study's age‐ and gender‐matched comparison. In addition, hypo‐IgG's beneficial impact eliminates any defect in IgG production or the local availability of antibodies in bronchial lumen. In CF, the thick and sticky mucus is due to the reduction in the airway surface's liquid depth and composition, which is a consequence of a defective CFTR Cl− channel function and ENaC Na+ channel hyperactivity, both of which contribute to CF's lung pathogenesis.^19^ These abnormalities of the mucus could prevent the access of antigens across the mucosal barrier, thus reducing systemic immune stimulation in some patients with CF.^3^, ^6^ A CFTR‐related intrinsic defect of the immune response that particularly affects the innate inflammatory response has similarly been suggested.^20^, ^21^ Finally, the link between mutations in the CFTR gene and disease manifestations is not yet completely understood. In addition to CFTR, in patients with CF, there are other genetic factors called “modifier genes” that modulate a patient's pathology and course.^12^, ^22^, ^23^, ^24^ We therefore suggest that modifier genes that would impact IgG status could explain the more favorable respiratory clinical course of CF patients with hypo‐IgG. Due to the gradual physiological and age‐related increase in immunoglobulin concentrations in the serum, Serone et al.^18^ hypothesized that the hs1.2 variant, which is one of the enhancers that modulate the Ig heavy chain expression, may impact the IgG levels in healthy children in different ways. These authors have demonstrated that hs1.2 polymorphism in IgG expression plays a fundamental role in children but not in adult populations, and that this is independent of the number of circulating mature B cells.^19^ Oxelius et al.^25^ showed that Gm allotypes experience different maturation rates during childhood. Gm allotypes are genetic variants of the heavy IgG chains, which are found in all IgG subclasses, except for IgG4. They exert an influence over IgG subclass levels. G2m(23) concerns the IgG2 subclass, which is the most abundant after IgG1. Children that are negative for G2m(23) experience a relative delay in maturation, which is expressed by lower IgG2 levels. A decrease in these IgG2 levels could influence their total IgG levels.^25^ In CF, low levels of IgG2 and of the antibodies to *Hemophilus influenzae* and *Streptococcus pneumoniae* have been described. However, they were not associated with any decline in clinical well‐being.^3^ Deciphering the genetic basis and molecular signaling pathways should improve our understanding of the potential mechanisms underlying this more favorable respiratory clinical course for CF patients with hypo‐IgG.

Compared with previous studies, no hyper‐IgG has been highlighted in the current work. The hyper‐IgG described in CF is the consequence of continuous antigenic stimulation on account of chronic respiratory infection and inflammation responsible for the progressive lung destruction. This has, therefore, been reported in older CF patients and is associated with a worse clinical course and lung function.^1^, ^3^, ^4^ Indeed, it is widely agreed that CF patients’ lung is a particular niche that enables certain bacteria, such as *PA*, to switch to the mucoid phenotype and overexpress exopolysaccharide alginate, which protects against certain antibiotics, reduces polymorphonuclear chemotaxis, inhibits complement activation, and decreases phagocytosis by neutrophils and macrophages. Despite a potent antibody response to *PA*, patients with CF fail to clear this infection.^12^, ^26^, ^27^ As in the case of hypo‐IgG, CF patients with hyper‐IgG can display paradoxical outcomes. In recent decades, CF therapy has been instrumental in significantly improving both the patients’ quality of life and life expectancy. Given that this progress in CF therapy comprises the aggressive treatment of any lung infection, this could decrease chronic inflammation, thereby modifying the IgG profile of pediatric patients with CF.

One limitation to our investigation is the retrospective nature of this study, along with some missing data including IgG values. We may, thus, have underestimated the transient IgG deficiency. In addition, we chose to convert IgG levels into Z‐scores, thus rendering it more difficult to compare our percentages with those of other studies. We are, nevertheless, convinced that this is the best way to handle and interpret IgG levels in the pediatric population.

## 5 CONCLUSIONS

In recent years, the IgG profile of pediatric patients with CF has changed, especially concerning hyper‐IgG. This is likely to be explained by that optimal management now comprises the aggressive treatment of lung infection. In a significant portion of the pediatric CF population, hypo‐IgG is transient and only identifiable using longitudinal assessments. Therefore, three populations can be identified, according to the patient's IgG profile during their follow‐up since diagnosis: a first with persistent hypo‐IgG since diagnosis; a second with a transient deficiency, which likely normalizes in adolescence; a third whose IgG remains within a normal range. CF patients with hypo‐IgG appear to be protected from PEx and therefore could present a more favorable respiratory course, even with low IgG levels since diagnosis. This suggests that IgG levels could be a useful outcome marker in CF patient follow‐up to predict their respiratory clinical course.

## CONFLICT OF INTERESTS

The authors declare that the research was conducted in the absence of any commercial or financial relationships that could be construed as a potential conflict of interest.

## Supporting information

Supporting information.

## Data Availability

The data that support the findings of this study are available from the corresponding author upon reasonable request.
