# Supplementary material for: Immunoglobulin G: A useful outcome marker in the follow‐up of cystic fibrosis patients?
Source: Immun Inflamm Dis. 2021 Mar 30;9(2):608–14. doi: 10.1002/iid3.426 (PMC8127551; doi:10.1002/iid3.426)
Supplement: Supplementary file 1 — Supporting information. [file IID3-9-608-s001.docx]

Table. Algorithms used to consider the variations of age-related reference values and definition of Z-scores

| Age range (months) | equations for age (in months) matched log p50 IgG |
| --- | --- |
| 3.5 to 11.5 | log p50 IgG = 0,235 + 0,09555*age - 0,00429*(age)² |
| 11.5 to 18 | log p50 IgG= 0,7134 - 0,002281*age + 0,0006397*(age)² |
| 18 to 156 | log p50 IgG = 0,8474 + 0,002296*age - 7,812e-06*(age)² |
| 156 to 340 | log p50 = 1,008 + 8,056e-05*age - 9,89e-08*(age)² |
|  |  |
| 4 to 240 | log SD IgG = 0,1055 - 0,0002045*age + 3,711e-07*(age)² |
| Definition-convention |  |
| Z-scores IgG† | = (log value – log p50 f(age)) / log SD f(age) |

†conventional formula used with log transformed data
